# A Hairy Tail of Rapunzel Syndrome: A Video Case Report of the Laparoscopic Management of a Giant Trichobezoar Causing Gastric Outlet Obstruction

**DOI:** 10.7759/cureus.117872

**Published:** 2026-10-13

**Authors:** Sajid Malik, Samina Alim, Chun Pui Joshua Wong, Nicholas Nicholas Davison, Francesco DiMaggio

**Affiliations:** 1 General Surgery, Croydon Health Services NHS Trust, Croydon, GBR; 2 General Surgery, Croydon University Hospital, London, GBR; 3 Gastroenterology, Croydon University Hospital, London, GBR

**Keywords:** case report, gastric outlet obstruction, giant trichobezoar, laparoscopic surgery, rapunzel syndrome

## Abstract

Trichobezoars are rare gastrointestinal concretions formed by accumulated ingested hair, occurring predominantly in young women with trichotillomania and trichophagia. Extension beyond the pylorus into the small bowel defines Rapunzel syndrome, which may present with gastric outlet obstruction. We report a 25-year-old woman with a three-year history of progressive weight loss, early satiety, postprandial vomiting, and iron-deficiency anaemia. Oesophago-gastro-duodenoscopy and computed tomography revealed a giant gastric trichobezoar extending to the distal duodenum. After two unsuccessful endoscopic extraction attempts, laparoscopic gastrotomy with en bloc extraction of a 38-cm, 220-g trichobezoar (weighed without the retrieval bag or container) with a 14-cm duodenal tail was performed. Recovery was uneventful; postoperative contrast study showed no leak or obstruction. At three months, the patient was asymptomatic, gaining weight, and engaged in cognitive behavioural therapy. Laparoscopic gastrotomy is feasible for giant trichobezoars when adequate exposure and contained extraction can be achieved. Concurrent psychiatric assessment and ongoing psychological care are essential to prevent recurrence.

## Introduction

Trichobezoars are rare intraluminal gastrointestinal masses formed by the accumulation of ingested hair, most commonly within the stomach [1]. They predominantly affect young women and are frequently associated with trichotillomania (compulsive hair-pulling) and trichophagia (hair-eating) [2,3]. Because hair is indigestible and compacts within the gastric folds, a trichobezoar may enlarge gradually over months to years, often remaining unrecognised until symptoms develop. 

Clinical presentation is usually insidious and non-specific, with abdominal pain, nausea, vomiting, early satiety, weight loss, iron-deficiency anaemia, or malnutrition [4]. Extension beyond the pylorus into the duodenum or small bowel is known as Rapunzel syndrome. This uncommon variant may cause gastric outlet obstruction, small-bowel obstruction, perforation, intussusception, or peritonitis [1,4].

Contrast-enhanced computed tomography of the abdomen and pelvis (CTAP) is the imaging modality of choice for evaluating bezoar size, composition, and distal extent. Classic CT findings include a heterogeneous, mottled-appearing intraluminal mass (reflecting a mixture of retained ingested material and trapped air, rather than contrast enhancement) with trapped air bubbles and a well-defined gastric or duodenal outline [5]. CTAP can also identify the complications described above, as well as separate distal bezoars [5,6].

Oesophago-gastro-duodenoscopy (OGD) complements CT by providing direct visualisation of the hair mat, confirming the diagnosis, assessing associated mucosal injury (gastritis, ulceration, duodenitis), and determining the feasibility of endoscopic removal [6]. The combination of CTAP and OGD is essential for accurate preoperative planning and guides the decision between endoscopic and surgical management [5,6].

Diagnosis is often delayed as patients may not voluntarily disclose hair-pulling or hair-eating behaviours and may have no visible alopecia. Rapunzel syndrome should be suspected in young patients, particularly women, presenting with gastric outlet obstruction or chronic unexplained upper gastrointestinal symptoms. The classic triad includes a young female patient, a palpable epigastric mass, and iron-deficiency anaemia, though this is not always present [1,2,5]. As a history of trichotillomania or trichophagia is rarely volunteered, direct, non-judgemental questioning is essential [4]. In patients with gastric outlet obstruction of unclear aetiology, trichobezoar should be considered in the differential diagnosis, as early recognition can prevent serious complications [1,4].

Physical examination may be normal or reveal an epigastric mass, while laboratory abnormalities generally reflect chronic blood loss, reduced intake, or malnutrition rather than a specific diagnostic pattern. Although a careful behavioural history is useful, imaging and endoscopy remain central to diagnosis and operative planning [3-6].

Management strategy depends on bezoar size, extent, and composition. Small trichobezoars may be amenable to endoscopic removal, whereas large, densely compacted masses usually require surgery. Open gastrotomy has traditionally provided direct access for controlled en bloc extraction in Rapunzel syndrome, although successful laparoscopic management has rarely been reported [2,7]. The laparoscopic approach remains uncommon because of several technical challenges, including the risk of peritoneal contamination from spillage of gastric or intestinal contents during extraction, the difficulty of manipulating a large, heavy, solid intraluminal mass through limited laparoscopic ports, and the technical demands of safely delivering a duodenal or jejunal extension without mucosal injury, perforation, or incomplete removal. These considerations must be weighed against the morbidity of open laparotomy when selecting the operative approach [7].

We describe a case of laparoscopic gastrotomy with intact extraction of a giant trichobezoar causing gastric outlet obstruction, accompanied by an operative technical video. This report is presented in accordance with the CAse REport (CARE) guidelines.

## Case presentation

Presentation and investigations

A 25-year-old woman underwent outpatient OGD to investigate a three-year history of progressive weight loss and early satiety, with more recent postprandial vomiting and solid-food dysphagia. The dysphagia was attributed to distal oesophageal dilatation secondary to chronic gastric outlet obstruction.

Her medical history included irritable bowel syndrome, sickle cell trait, and iron-deficiency anaemia managed with oral iron supplementation. A history of trichotillomania and trichophagia was not volunteered at presentation, and no visible alopecia was noted on examination; the diagnosis was established later through directed psychiatric assessment. In the preceding months, she developed intermittent abdominal pain, vomiting, and dyspepsia, with unintentional weight loss of approximately 15 kg (height 1.64 m; pre-illness weight 62 kg, BMI 23.0 kg/m²; weight at admission 47 kg, BMI 17.5 kg/m²). She had no prior abdominal surgery. Although some symptoms were longstanding, the recent onset of vomiting and accelerated weight loss raised concern for a mechanical obstructive cause. There was no clinical evidence of peritonitis or perforation.

OGD revealed a giant trichobezoar occupying the entire gastric lumen (Figure 1A). Attempts to manipulate the mass with endoscopic forceps were unsuccessful due to its size and density, and the patient was admitted for further management. She remained haemodynamically stable throughout; blood tests were unremarkable apart from pre-existing microcytic anaemia.

**Figure 1:**
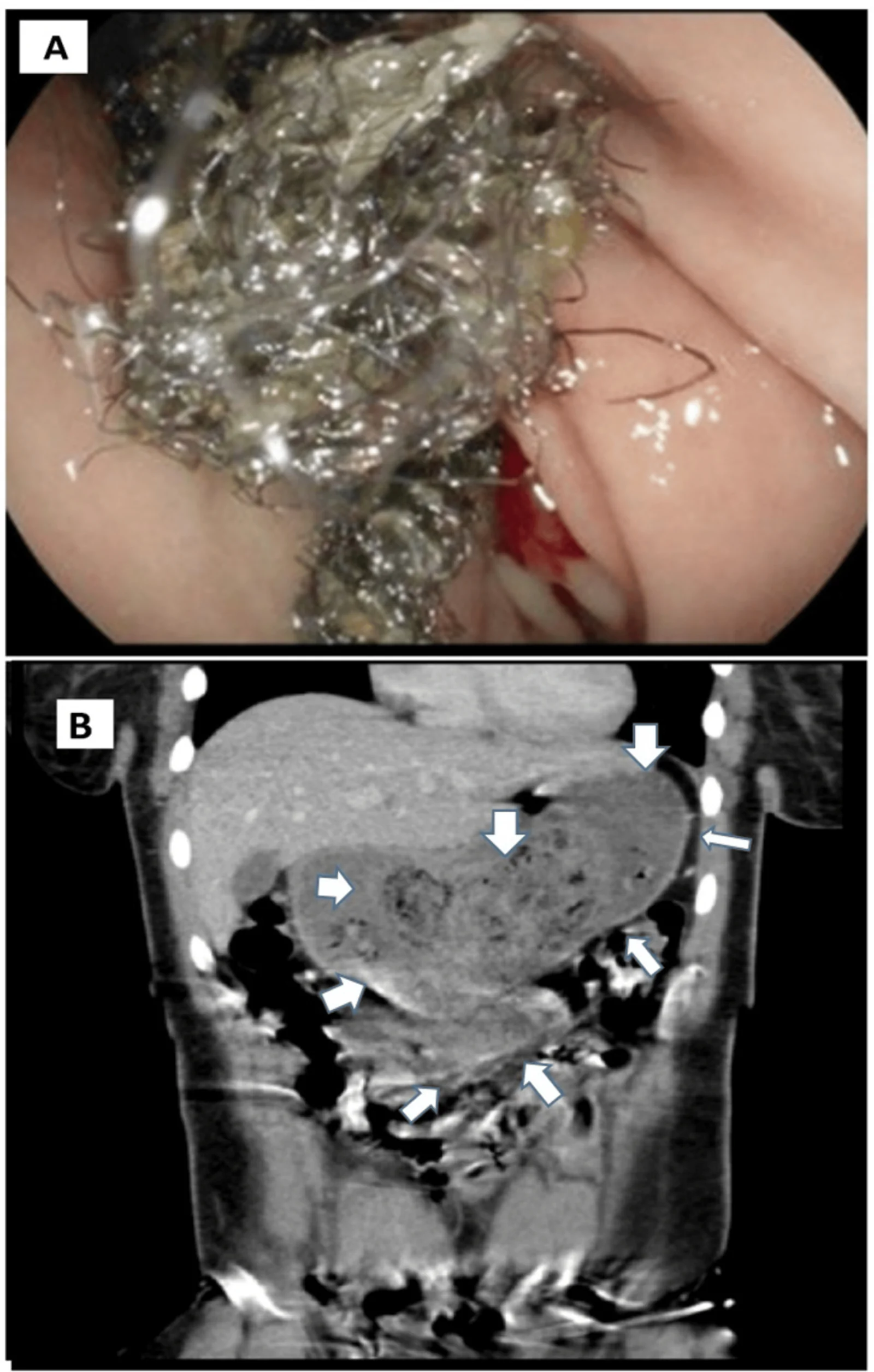
Diagnostic findings. (A) Oesophago-gastro-duodenoscopy shows a giant trichobezoar occupying the gastric lumen. (B) Contrast-enhanced computed tomography demonstrates a distended stomach, marked with arrows, containing a large intraluminal hair-like mass extending beyond the pylorus into the duodenum.

CTAP demonstrated a distended stomach containing a large hair-density intraluminal mass extending into the distal duodenum, consistent with Rapunzel syndrome causing gastric outlet obstruction. Mild distal oesophageal dilatation was present, without perforation (Figure 1B).

Laboratory evaluation revealed microcytic, hypochromic anaemia (haemoglobin 8.2 g/dL (reference range: 11.5-15.5 g/dL), mean corpuscular volume (MCV) 68 fL (reference range: 76-96 fL)) with iron deficiency confirmed by severely depleted ferritin (4 μg/L (reference range: 15-150 μg/L)) and low transferrin saturation (6% (reference range: 15-50%)). Mild hypoalbuminaemia (32 g/L (reference range: 34-48 g/L)) indicated protein-energy malnutrition secondary to reduced oral intake. Normal inflammatory markers (CRP <5 mg/L (reference range: <5 mg/L)) and faecal calprotectin (38 μg/g (reference range: <50 μg/g)) effectively excluded inflammatory bowel disease, while negative coeliac serology (tissue transglutaminase-immunoglobulin A (tTG-IgA) <1 U/mL (reference range: <7 U/mL)) ruled out coeliac disease. These findings supported a mechanical gastrointestinal pathology as the primary cause of her symptoms, with iron deficiency and malnutrition as secondary consequences of the trichobezoar rather than a primary inflammatory or autoimmune process.

Psychiatric assessment, conducted using the Massachusetts General Hospital Hairpulling Scale (MGH-HPS), identified moderate trichotillomania and trichophagia dating back to adolescence, with the most recent episode occurring one month before presentation. The patient scored 14/21 on the MGH-HPS, indicating clinically significant symptoms. Mental state examination was otherwise unremarkable, with no evidence of comorbid anxiety, depression, or obsessive-compulsive disorder.

A further endoscopic extraction attempt was undertaken under general anaesthesia in the operating room. The patient had been consented for OGD with a view to endoscopic extraction, with additional consent for laparoscopic gastrotomy and possible open conversion should endoscopic removal prove unsuccessful. The trichobezoar was found to extend from the gastro-oesophageal junction to the fourth part of the duodenum, with associated duodenitis. Various techniques were attempted, including snare fragmentation, polypectomy snare mobilisation, and suction retrieval; however, mobilisation was unsuccessful due to the density and adherence of the mass. The procedure was abandoned due to the risk of oesophageal or gastric perforation. Laparoscopic extraction was then performed.

Surgical procedure

Video 1 demonstrates the surgical procedure.

**Video 1:** Laparoscopic gastrotomy and intact extraction of a giant trichobezoar in Rapunzel syndrome. The video demonstrates port placement, anterior gastrotomy, mobilisation of the gastric component and small-bowel tail, contained retrieval, and stapled gastrotomy closure with suture reinforcement and omental buttressing.

The patient underwent laparoscopic gastrotomy in the supine position with a split-leg configuration and reverse Trendelenburg positioning. A urinary catheter was inserted. Pneumoperitoneum was established with the open Hasson technique via a 10-mm supraumbilical camera port. Additionally, a 12-mm working port in the left paramedian region and a 5-mm working port in the right paramedian region were placed. 

Intraoperatively, the stomach was markedly distended, with a large intraluminal mass extending beyond the pylorus (Figure 2A). An anterior vertical gastrotomy was created in the gastric body and extended to approximately 12 cm. A densely compacted trichobezoar occupying the stomach and extending into the duodenum and proximal jejunum was identified (Figure 2B). Its cohesive, adherent nature and the limited luminal space prevented mobilisation with standard laparoscopic graspers.

**Figure 2:**
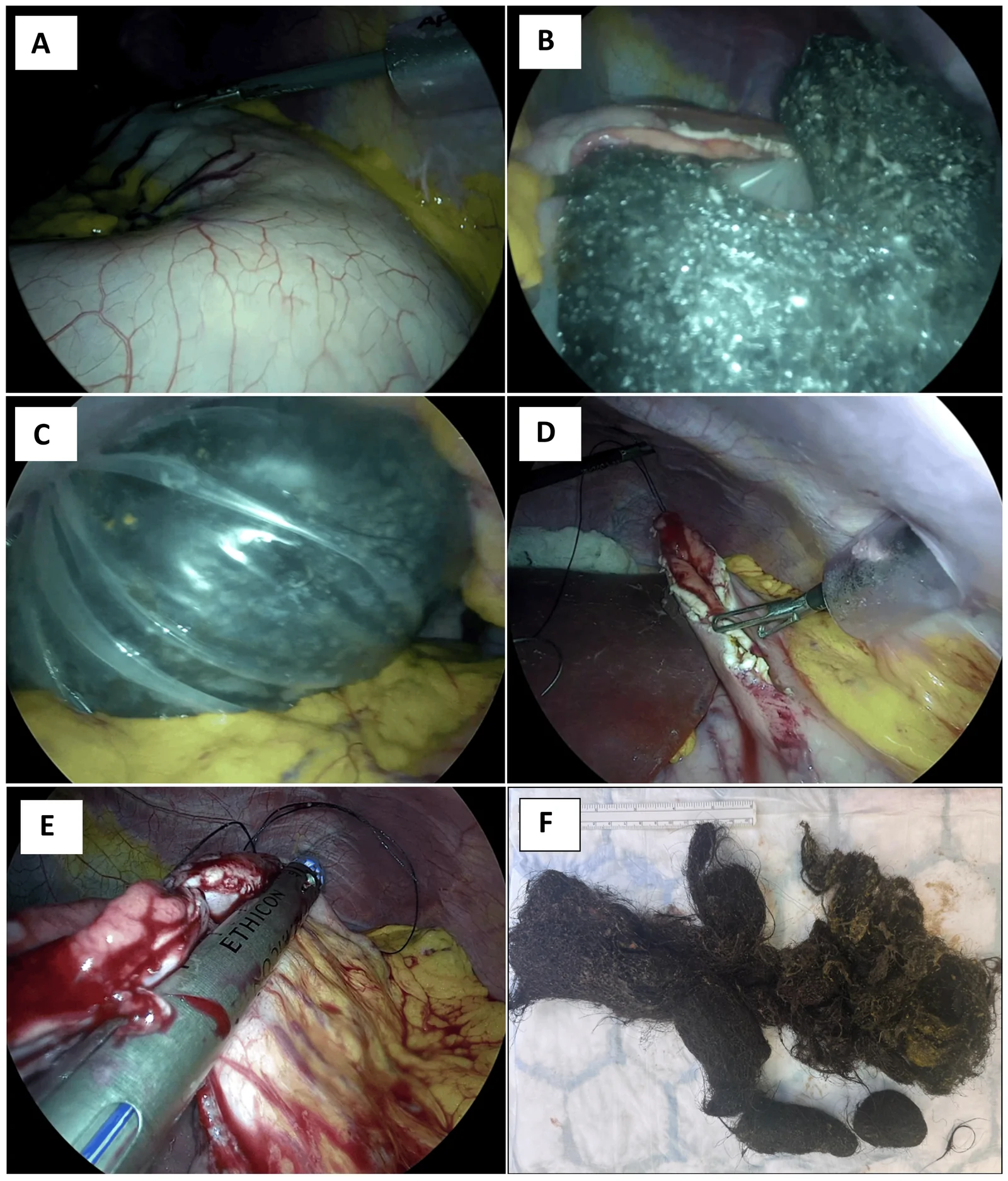
Laparoscopic extraction of the trichobezoar. (A) Markedly distended stomach containing the intraluminal mass. (B) Giant trichobezoar exposed through the anterior gastrotomy. (C) Controlled mobilisation and delivery of the specimen into a retrieval bag. (D, E) Stapled closure of the gastrotomy, reinforced with interrupted sutures. An omental buttress was applied after closure. (F) The extracted 220-g trichobezoar.

A large specimen retrieval bag was positioned beneath the stomach to contain the specimen and minimise intra-abdominal contamination. Using a heavy grasper, the proximal part of the bezoar was exteriorised through the gastrotomy. Clockwise rotation (rather than forceful withdrawal) of the mass released its tail from the duodenum and proximal jejunum. This allowed the direction of resistance to be assessed and reduced the risk of bowel injury or separation of a distal fragment, with direct placement into the retrieval bag (Figure 2C). Once the tail had been delivered through the pylorus, the cohesive nature of the bezoar permitted removal as a single specimen.

The umbilical fascial incision was extended to approximately 3 cm to facilitate specimen delivery. A wound protector was placed to protect the incision edges during extraction. During extraction, a small defect (approximately 0.5-1 mm) developed in the first retrieval bag, resulting in minor contamination confined to the umbilical wound without intraperitoneal spillage. A second large specimen retrieval bag was introduced, and the intact trichobezoar was successfully extracted. The wound was irrigated with normal saline and povidone-iodine, and no retained hair fragments were identified on careful inspection.

The gastrotomy was closed using three firings of a 60-mm linear stapler (Endo GIA™, Covidien, Minneapolis, Minnesota, United States) through a 12-mm left paramedian port, reinforced with interrupted 3-0 polydioxanone (PDS) sutures and an omental buttress (Figure 2D-2E). After closure, the staple line and abdominal cavity were carefully inspected for residual hair and contamination. A Robinson 24F drain was placed adjacent to the gastric staple line for postoperative monitoring. The specimen weighed 220 g (Figure 2F). The total operative time was 70 minutes, the estimated blood loss was 25 mL, and no intraoperative complications occurred.

Postoperative course

The postoperative course was uneventful. The patient's progress is summarised chronologically as follows. On day 0 (day of surgery), the patient returned to the surgical ward haemodynamically stable. Pain was managed with patient-controlled analgesia (PCA) using morphine. A nasogastric tube remained in situ overnight for gastric decompression. On day 1 (postoperative day 1), oral intake commenced with clear fluids (water, clear broth), which were well tolerated. The nasogastric tube was removed. Mobilisation commenced with physiotherapy assistance, and bowel sounds were audible, indicating early return of gastrointestinal motility.

On day 2 (postoperative day 2), the diet was advanced to full fluids (yoghurt, soup, custard), with no vomiting or abdominal distension. Analgesia was stepped down to oral paracetamol 1 g four times daily. Drain output was <50 mL of serous fluid. On day 3 (postoperative day 3), the diet was advanced to a soft diet (mashed potato, scrambled eggs), which was tolerated without symptoms. Drain output decreased to <30 mL. There was no fever or leukocytosis. On day 4 (postoperative day 4), the patient continued to tolerate a soft diet well. Stool was passed spontaneously, confirming satisfactory return of bowel function. The drain was removed after confirming minimal output (<5 mL/24 hours). On day 5 (postoperative day 5), a water-soluble contrast study (Gastrografin) was performed, demonstrating normal passage through the stomach and duodenum without leak, residual filling defect, or obstruction (Figure 3). No further abnormalities were identified. 

**Figure 3:**
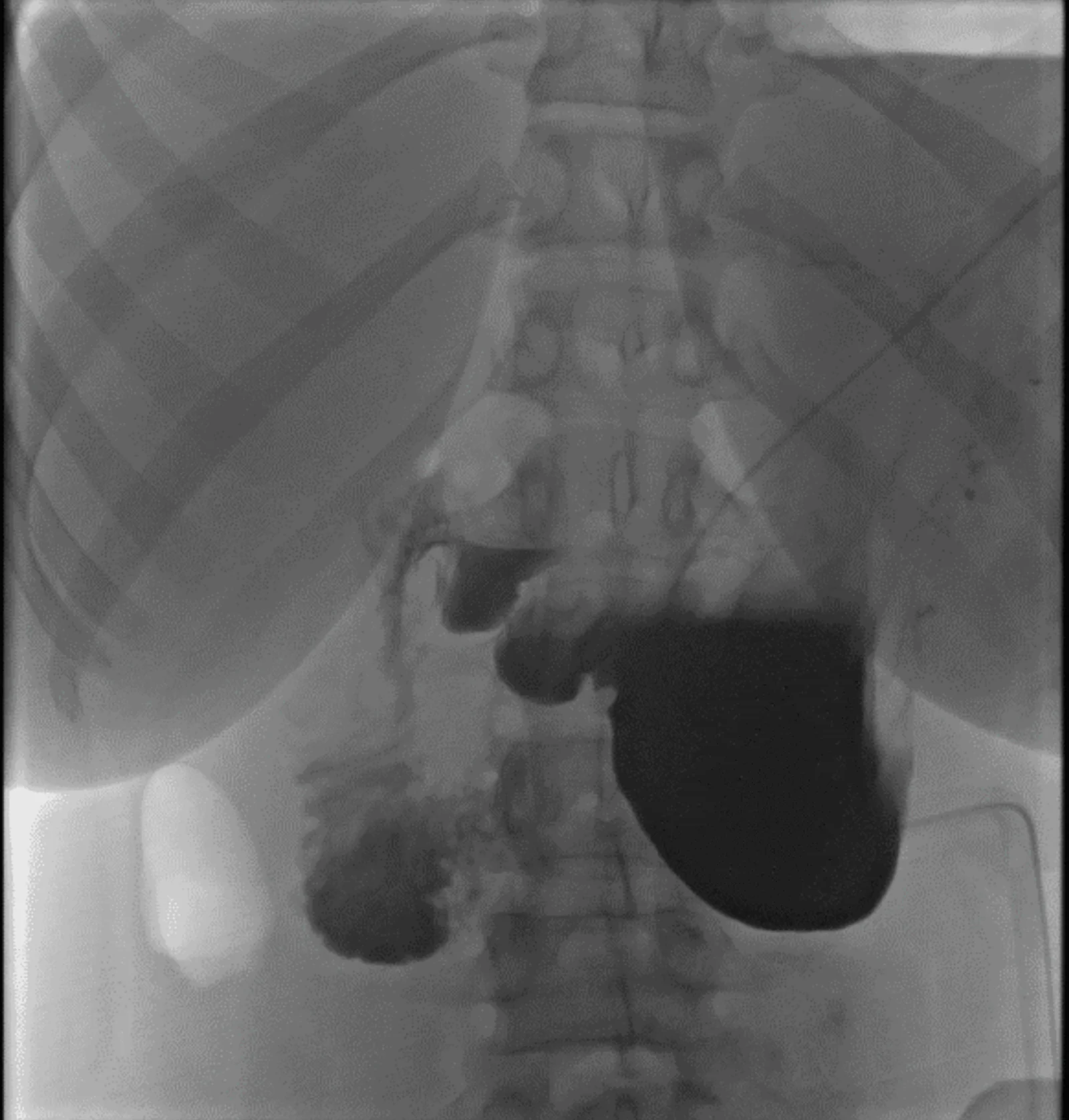
Postoperative water-soluble contrast radiograph. This study demonstrates normal gastric opacification and passage of contrast through the pylorus into the duodenum, without leak, residual filling defect, or obstruction.

On day 6 (postoperative day 6), the patient was discharged home with oral co-amoxiclav 625 mg three times daily for five days, prescribed in response to the retrieval bag rupture and potential wound contamination, together with a proton pump inhibitor (omeprazole 20 mg once daily for six weeks) for staple line protection and analgesia as required. Her pre-existing oral iron supplementation was continued. Community psychiatric follow-up and cognitive behavioural therapy (CBT) were arranged to address the underlying trichotillomania and trichophagia.

On day 15 (postoperative day 15), telephone follow-up confirmed the patient was eating well without symptoms. Wound healing was satisfactory per patient report. On day 30 (postoperative day 30), outpatient clinic review confirmed stable weight, tolerance of a normal diet, and absence of abdominal pain or vomiting. Haemoglobin had improved to 10.2 g/dL. Psychiatric follow-up was ongoing.

On day 90 (three months; final surgical follow-up and outcome), the patient remained asymptomatic, was eating normally, and was gaining weight, with BMI improving from 17.5 kg/m² at admission to 19.8 kg/m². She reported no early satiety or postprandial vomiting, in contrast to her preoperative symptoms. Iron-deficiency anaemia had resolved (haemoglobin 11.8 g/dL, ferritin 28 μg/L), reflecting improved dietary iron intake following relief of the obstruction; no additional postoperative iron supplementation was required beyond her pre-existing oral supplementation. Psychiatric follow-up consisted of community psychiatric review and CBT targeting trichotillomania and trichophagia, which she continued at three months. No pharmacotherapy was prescribed, as mental state examination revealed no comorbid anxiety, depression, or obsessive-compulsive disorder and symptom severity was moderate. She was discharged from surgical follow-up with instructions for continued psychiatric care and general practitioner monitoring.

## Discussion

Rapunzel syndrome is a rare, advanced manifestation of trichobezoar in which the gastric hair mass extends beyond the pylorus. Published experience remains dominated by case reports and small, predominantly paediatric series, and robust incidence data for trichobezoars and Rapunzel syndrome are lacking. Reported cases show a marked female preponderance and typically present in childhood, adolescence, or young adulthood, most commonly between the ages of five and 25 years; since its first reported description, systematic reviews of published cases have also found that male cases tend to present at a younger age [1,2,4,8]. Common manifestations include abdominal pain, nausea or vomiting, early satiety, weight loss, a palpable epigastric mass, anaemia, and nutritional or protein depletion [2,4,5,9]. Diagnosis may be delayed because hair accumulates gradually, early symptoms are absent or non-specific, and trichotillomania or trichophagia may not be disclosed or accompanied by visible alopecia [2-5]. Clinicians should specifically enquire about hair-pulling and hair-eating behaviours in a non-judgemental manner, as patients often feel shame and may not disclose these habits spontaneously [3,9]. The three-year symptomatic course, progressive weight loss, iron-deficiency anaemia, and mild hypoalbuminaemia in our patient are consistent with this insidious pattern, while the later development of vomiting reflected progression to gastric outlet obstruction.

CT and OGD provide complementary information. CT typically demonstrates a well-defined heterogeneous intraluminal mass with intermixed gas, while multiplanar imaging can define postpyloric extension, locate separate distal bezoars, and identify obstruction, perforation, or other complications [5,6]. A frequently cited CT series detected all surgically confirmed bezoars and was particularly useful for identifying additional gastrointestinal masses, but 16 of its 17 patients had phytobezoars; its diagnostic performance should therefore not be assumed to apply identically to trichobezoars [5]. OGD confirms the characteristic hair mat, assesses mucosal injury, and may test whether endoscopic mobilisation is feasible [6]. However, when a giant mass obstructs the pylorus or prevents duodenal intubation, OGD cannot reliably exclude a longer Rapunzel tail or satellite bezoars [8], as their size, density, and adherence to the gastric mucosa make fragmentation and retrieval challenging. Repeated manipulation may cause mucosal injury, while incomplete fragmentation can leave residual material that migrates distally and causes further obstruction [7,9-14]. In this case, OGD established the nature and density of the mass, whereas CT demonstrated its distal duodenal extension and excluded perforation; the combination was important for operative planning.

Endoscopic treatment is substantially more difficult for a trichobezoar than for many phytobezoars because hair is indigestible and becomes densely entangled into a cohesive cast. In a paediatric literature review published in 2010, only two of 40 attempted endoscopic removals were successful, although this historical, case-report-derived proportion is not an estimate of contemporary adult practice [2]. Reported fragmentation methods include electrohydraulic lithotripsy in plant-material gastric bezoars [10], holmium-doped yttrium aluminum garnet (Ho:YAG) laser fragmentation in gastric bezoars not shown to represent giant trichobezoars [11], a modified needle-knife with monopolar current for trichobezoars within a mixed-bezoar series [12], and snare fragmentation with electrocautery and argon plasma coagulation in an individual paediatric trichobezoar [13]. More recent cap-assisted endoscopic removal of a massive paediatric trichobezoar has also been described [14]. These reports demonstrate technical possibilities in selected patients, but evidence derived from phytobezoars or mixed-composition series does not establish efficacy for a giant trichobezoar with a long postpyloric tail. Repeated fragmentation may require multiple scope passages, with risks of mucosal injury, incomplete clearance, and distal migration; small-bowel obstruction after migration of trichobezoar fragments following endoscopic treatment has been reported [2,15]. After two unsuccessful attempts in our patient, further fragmentation of an immobile mass extending into the distal duodenum was judged less safe and less likely to achieve complete clearance than operative extraction.

Operative removal is generally required for giant, densely consolidated, or postpyloric trichobezoars. Open gastrotomy has historically been favoured because it permits wide exposure, direct palpation, intact extraction, and efficient examination of the stomach and small bowel for a continuous tail, satellite bezoars, or detached fragments; an enterotomy can be added when a distal component is impacted [2,9]. Laparoscopic removal has nevertheless been feasible in selected cases since the first report by Nirasawa et al. [16]. Described alternatives include laparoscopy-assisted exteriorisation of the stomach with temporary gastrocutaneopexy to limit peritoneal contamination [7] and laparoscopic gastrotomy with protected specimen retrieval [8]. Minimally invasive access may reduce the abdominal wall burden, although a very large specimen may still require an enlarged or auxiliary extraction incision, and intracorporeal fragmentation can prolong the procedure and increase the opportunity for hair or gastric-content spillage [2,8,16]. In a retrospective comparison of 24 patients with bezoar-induced small-bowel obstruction, Yau et al. reported shorter operative time, earlier return of bowel function, fewer postoperative complications, and shorter hospital stay with laparoscopic versus open surgery [17]. However, this series was not specific to giant gastric trichobezoars or Rapunzel syndrome, and evidence in this population remains limited to case reports and small series. A systematic review by Gorter et al. identified only 20 reported cases of laparoscopic management of trichobezoars, with most managed by open gastrotomy; among the laparoscopic cases, operative times ranged from 45 to 120 minutes and hospital stay from two to seven days, comparable to our experience [2,7,8]. In our case, the 70-minute operative time compares favourably with reported open series, in which operative times typically range from 90 to 150 minutes [2,3,7]. The six-day hospital stay, however, should be interpreted with caution: it was longer than might be expected for an uncomplicated laparoscopic gastrotomy and was influenced by the delayed day 5 contrast study and the retrieval bag rupture, rather than by the operative approach itself. Had the contrast study been performed earlier and had no bag rupture occurred, discharge may have been achieved sooner. Comparisons of length of stay between approaches should therefore account for such case-specific factors. No high-quality comparative evidence establishes that laparoscopy is superior to open gastrotomy for giant gastric trichobezoar or Rapunzel syndrome, and selection should reflect the size and mobility of the mass, distal extent, complications, abdominal domain, and surgeon experience. 

The rotational extraction technique employed in our case represents a technical refinement that may expand the applicability of laparoscopic management. By rotating the bezoar rather than applying traction, the tail can be extracted from the duodenum with reduced risk of mucosal injury or fragmentation [7,8]. This technique is demonstrated in Video 1 at 01:47 (min:sec). This approach has been described in open surgery but has not been widely reported in laparoscopic series [2]. Contained extraction using a retrieval bag is another important technical consideration, minimising intra-abdominal contamination and reducing the risk of retained hair fragments [7,8]. These observations support the potential advantages of minimally invasive treatment but cannot be extrapolated directly to all cases of giant gastric trichobezoars or Rapunzel syndrome. Evidence specific to this condition remains limited to case reports and small series and does not establish the comparative superiority of either approach.

The potential consequences of delayed or incomplete treatment extend beyond gastric outlet obstruction. Pressure and mucosal irritation may cause gastritis, ulceration, bleeding, and iron-deficiency anaemia; in severe cases, gastric ulceration may progress to perforation, which carries a mortality rate of up to 30% in reported series [1,4]. Small-bowel obstruction may occur if fragments separate and migrate distally, particularly during attempted endoscopic extraction [2,15]. Intussusception, though rare, has also been reported [1]. These possibilities support complete removal and inspection for a residual tail or separate fragments. Our patient had marked iron-deficiency anaemia and nutritional compromise but no perforation, and both haematological and nutritional recovery followed relief of the obstruction.

Mechanical removal does not treat the behaviour that produced the trichobezoar. Trichotillomania is classified among obsessive-compulsive and related disorders, but its clinical and treatment profile is distinct from obsessive-compulsive disorder [18]. Trichophagia occurs in a subset of patients with trichotillomania and has been associated with greater hair-pulling severity, although the probability that an individual with trichophagia will develop a trichobezoar remains uncertain [19]. Screening should include depression, anxiety, obsessive-compulsive disorder, attention-deficit/hyperactivity disorder, and other clinically relevant comorbidities, all of which have been reported among adults with trichotillomania [19].

CBT with habit reversal training (HRT) represents the first-line psychological intervention for trichotillomania and trichophagia [18,20]. HRT involves awareness training to identify hair-pulling triggers and competing responses to replace the behaviour. Acceptance and commitment therapy (ACT) has also shown promise in maintaining long-term behavioural change [20]. Pharmacological options, including selective serotonin reuptake inhibitors (SSRIs) and N-acetylcysteine, may be considered as adjunctive therapy in refractory cases, though evidence for their efficacy in trichotillomania is limited [20].

Mechanical removal does not treat the behaviour that produced the trichobezoar, and multidisciplinary follow-up is therefore essential. Successful outcomes require collaboration between surgeons, gastroenterologists, psychiatrists, psychologists, and dietitians, with early psychiatric involvement ideally during the index admission so that behavioural therapy is initiated before discharge [2,3,5,9]. Regular follow-up, typically at monthly intervals initially, allows behavioural progress to be monitored and therapeutic strategies adjusted. Recurrent Rapunzel syndrome is documented, including after previous operative removal when behavioural follow-up was not maintained, although published reports specific to this variant are too sparse to support a reliable recurrence estimate [2]. Data from the broader trichobezoar literature suggest recurrence rates of approximately 10-20% without psychiatric follow-up, compared with less than 5% with ongoing psychological treatment, and a median time to recurrence of 12-24 months, although recurrence may occur as early as 3-6 months postoperatively [2,4]. These figures should be interpreted with caution given their heterogeneity and lack of specificity to Rapunzel syndrome. Family support is also important; family members can assist in monitoring for continued hair-pulling, providing reminders for behavioural therapy, and supporting dietary changes [4]. In paediatric patients, parental involvement is particularly important, as the condition may be associated with school difficulties or social isolation [2]. Our patient's engagement with CBT at three months is encouraging, although longer follow-up is required to assess durable behavioural control and recurrence.

Future research should focus on prospective studies comparing laparoscopic and open approaches for giant trichobezoars, with particular attention to patient selection criteria, operative outcomes, and long-term recurrence rates. The development of standardised surgical algorithms for bezoar management would be valuable, given the rarity of the condition and the lack of high-level evidence [2,7]. Innovations in endoscopic technology, including mechanical lithotripters and laser fragmentation, may expand the role of non-surgical management in selected cases [10-14]. Advances in psychiatric therapeutics, including novel pharmacotherapies targeting the glutamatergic system (such as N-acetylcysteine) and transcranial magnetic stimulation for refractory trichotillomania, may improve outcomes for the underlying behavioural disorder [20].

Limitations

This is a single case and cannot establish comparative superiority between laparoscopic and open surgery. The result should not be extrapolated to all presentations, particularly where perforation, peritonitis, or another complication requires wider exploration. The rarity of Rapunzel syndrome and the limited published experience with laparoscopy preclude firm conclusions about the optimal operative approach. Additionally, follow-up was limited to three months, which is insufficient to assess long-term recurrence rates.

## Conclusions

Rapunzel syndrome is a rare cause of gastrointestinal obstruction. Laparoscopic gastrotomy can permit controlled en bloc removal of a giant trichobezoar and is a feasible minimally invasive option when adequate exposure and contained extraction can be achieved. Definitive surgical management should be accompanied by psychiatric assessment and continuing psychological care to reduce recurrence.
